# P-972. Enhancing Antimicrobial Stewardship Efficiency: Approach to Building an Optimized Patient Alert List in the Electronic Health Record

**DOI:** 10.1093/ofid/ofaf695.1171

**Published:** 2026-01-11

**Authors:** Sarah B Green, Kristen Paciullo, Drunell Bailey, Sujit Suchindran

**Affiliations:** Emory University Hospital, Atlanta, Georgia; Emory Healthcare, Atlanta, GA; Emory Healthcare, Atlanta, GA; Emory University School of Medicine, Atlanta, GA

## Abstract

**Background:**

Prospective audit and feedback remains a core activity for antimicrobial stewardship (AS) programs (ASP). Increasingly robust regulatory requirements for ASPs also require institutional guideline development, data tracking, reporting, and education. Despite these increased requirements, ASPs may not experience a similar increase in resources. Many ASPs have turned to technology to increase AS workflow efficiency. However, foundation electronic health record (EHR) builds are often geared towards basic AS interventions and do not meet the needs of a regulatory-ready ASP team. Without optimization of foundational EHR workflows, ASPs must utilize alternative AS methods which are often time consuming and lack targets for institution-specific, high-risk populations. Here we describe our process in developing an optimized AS patient list to meet the needs of a large health-system with both academic and community hospitals.

**Figure:**
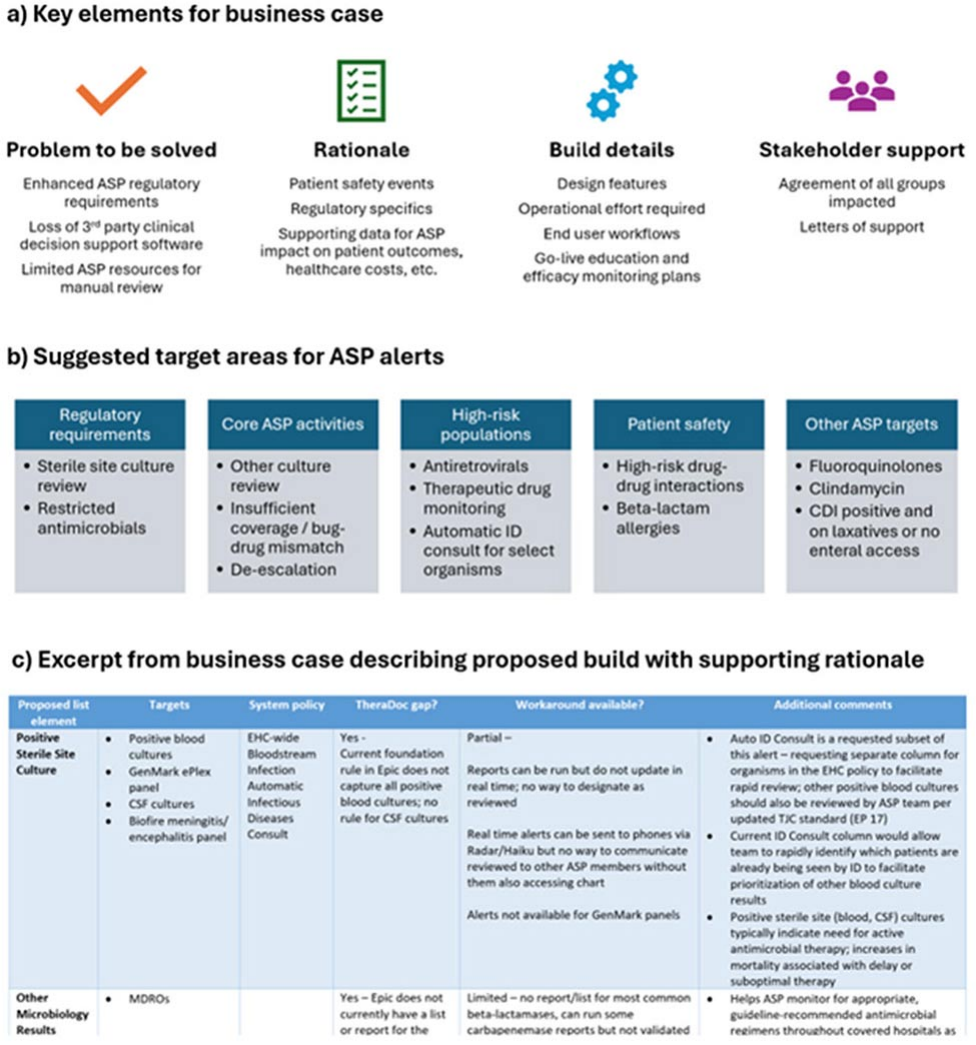
Key components for an ASP patient list business case, recommended targets for list build, and detailed view of build proposal. Abbreviations: ASP = antimicrobial stewardship program; CDI = C. difficile infection; CSF = cerebral spinal fluid; EHC = Emory Healthcare; EP = elements of performance; ID = Infectious diseases; TJC = The Joint Commission

**Figure:**
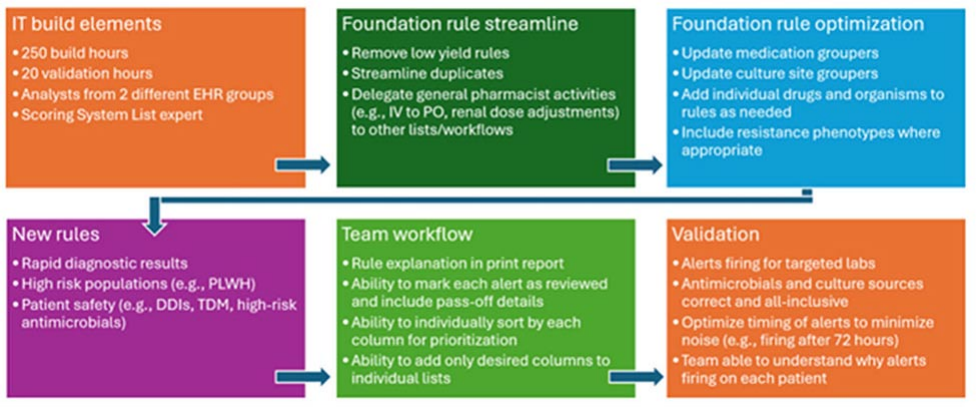
Process of patient list build. Abbreviations: DDI = drug-drug interaction; IT = information technology; IV = intravenous; PLWH = persons living with HIV; PO = by mouth; TDM = therapeutic drug monitoring

**Methods:**

The system-wide ASP team developed a business case describing the limited functionality of the foundation AS EHR build, an outline of the requested build based on key ASP targets, and the rationale for dedicated information technology (IT) resources (Figure 1). Once approved, the ASP project leads worked closely with the IT team to build and validate the patient list (Figure 2). The entire process took approximately 250 hours of IT build time and 20 hours of ASP time to complete. To assess initial improvements in ASP efficiency, overall numbers of AS interventions were measured before and after go-live.

**Figure:**
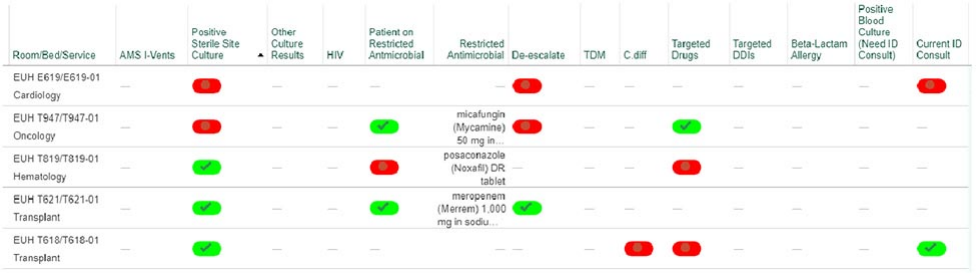
Detailed view of ASP patient list. Abbreviations: AMS = antimicrobial stewardship; C. diff = C. difficile infection; DDI = drug-drug interaction; ID = infectious diseases; HIV = Human Immunodeficiency Virus; TDM = therapeutic drug monitoring

**Figure:**
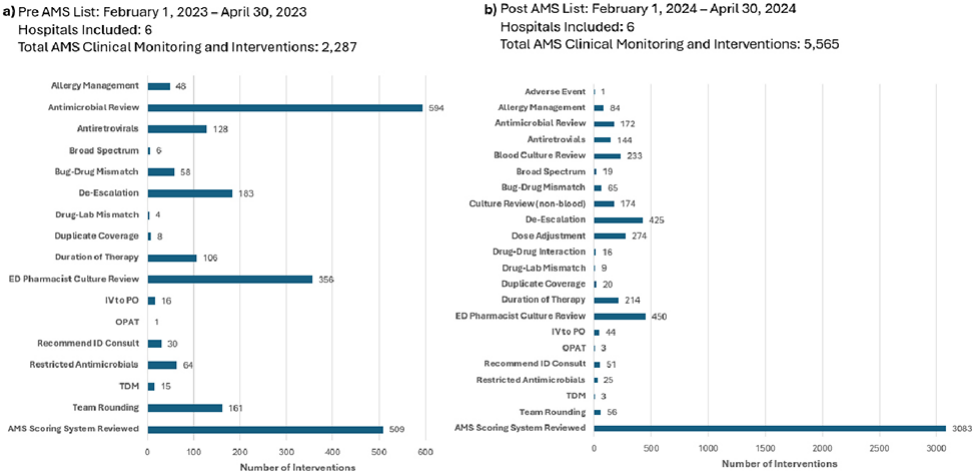
Summary of antimicrobial stewardship interventions before and after ASP patient list go-live. Abbreviations: AMS = antimicrobial stewardship; ASP = antimicrobial stewardship program; ID = infectious diseases; IV = intravenous; OPAT = outpatient parenteral antimicrobial therapy; PO = by mouth; TDM = therapeutic drug monitoring

**Results:**

An optimized AS patient list was created (Figure 3). Hospital-specific ASPs can customize the list through selection of individual alert columns and prioritize by sorting. In addition, the AS patient list allows communication within ASP teams by checking off individual alerts when reviewed and leaving pass-off within the print report. Despite no changes in ASP staffing resources, AS interventions increased by more than 240% after implementation (Figure 4).

**Conclusion:**

Over 270 IT and ASP hours were utilized to create an optimized ASP patient list in the EHR. AS interventions increased significantly without an increase in ASP staffing. Despite the upfront time costs, optimization of AS activities performed in the EHR can improve overall ASP efficiency.

**Disclosures:**

All Authors: No reported disclosures

